# The Evolution of Therapies in Non-Small Cell Lung Cancer

**DOI:** 10.3390/cancers7030864

**Published:** 2015-09-09

**Authors:** Vishal Boolell, Muhammad Alamgeer, David N. Watkins, Vinod Ganju

**Affiliations:** 1 Department of Medical Oncology, Monash Medical Centre, 823-865 Centre Road, East Bentleigh VIC 3165, Australia; E-Mails: alamgeer.muhammad@monash.edu (M.A.); vg@paso.com.au (V.G.); 2 Hudson Institute of Medical Research, Monash University, 27-31 Wright Street, Clayton VIC 3168, Australia; E-Mail: n.watkins@garvan.org.au; 3 Garvan Institute of Medical Research, 384 Victoria Street, Darlinghurst, Sydney NSW 2010, Australia; 4 UNSW Faculty of Medicine, St Vincent’s Clinical School, 390 Victoria Street, Darlinghurst, Sydney NSW 2010, Australia; 5 Department of Thoracic Medicine, St Vincent’s Hospital, 390 Victoria Street, Darlinghurst, Sydney NSW 2010, Australia

**Keywords:** non-small cell lung cancer (NSCLC), epidermal growth factor receptor (EGFR), anaplastic lymphoma kinase (ALK), tyrosine kinase inhibitors, immuno-oncology, cytotoxic T lymphocyte antigen-4 (CTLA-4), programmed death-1 receptor (PD-1), PD-1 inhibitors

## Abstract

The landscape of advanced non-small lung cancer (NSCLC) therapies has rapidly been evolving beyond chemotherapy over the last few years. The discovery of oncogenic driver mutations has led to new ways in classifying NSCLC as well as offered novel therapeutic targets for anticancer therapy. Targets such as epidermal growth factor receptor (EGFR) mutations and anaplastic lymphoma kinase (ALK) gene rearrangements have successfully been targeted with appropriate tyrosine kinase inhibitors (TKIs). Other driver mutations such as ROS, MET, RET, BRAF have also been investigated with targeted agents with some success in the early phase clinical setting. Novel strategies in the field of immune-oncology have also led to the development of inhibitors of cytotoxic T lymphocyte antigen-4 (CTLA-4) and programmed death-1 receptor (PD-1), which are important pathways in allowing cancer cells to escape detection by the immune system. These inhibitors have been successfully tried in NSCLC and also now bring the exciting possibility of long term responses in advanced NSCLC. In this review recent data on novel targets and therapeutic strategies and their future prospects are discussed.

## 1. Introduction

Lung cancer is the most common cancer worldwide as well as the leading cause of cancer related deaths [1]. Non-small cell lung cancer (NSCLC) accounts for up to 85% of all lung cancers and of these, adenocarcinoma and squamous cell carcinoma (SCC) account for 50% and 30% respectively [2,3]. Most of these patients present with locally advanced inoperable or metastatic disease, which makes their cancer incurable, and unfortunately almost all of these patients will die from their illness [4]. Despite multiple advances in the staging, diagnostic procedures and therapeutic options, the overall outlook has not greatly changed for the majority of patients with the overall 5-year survival having only marginally increased over the last decade from 15.7% to 17.4% [5].

**Figure 1:**
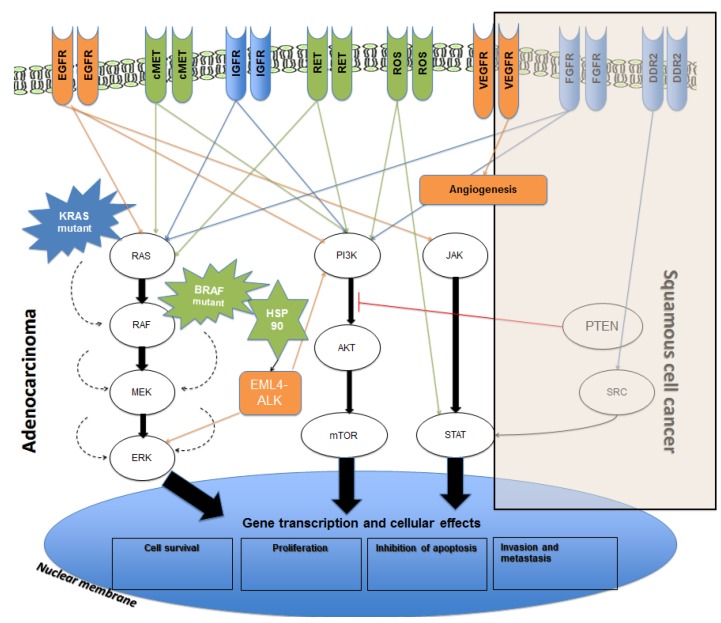
Overview of molecular pathways and potential targets in non-small lung cancer (NSCLC). Genetic targets with approved therapeutic agents are shown in orange. Targets with agents under development are shown in green while targets with no currently available inhibitors are shown in blue. Arrows represent the downstream activation with red line representing the inhibitory action. Dashed arrows represent the proposed auto-activatory mechanism of mutant K-RAS and BRAF (6). Vascular Endothelial Growth Factor (VEGF) activation occurs in both adenocarcinoma and squamous cell carcinoma (SCC).

**Figure 2:**
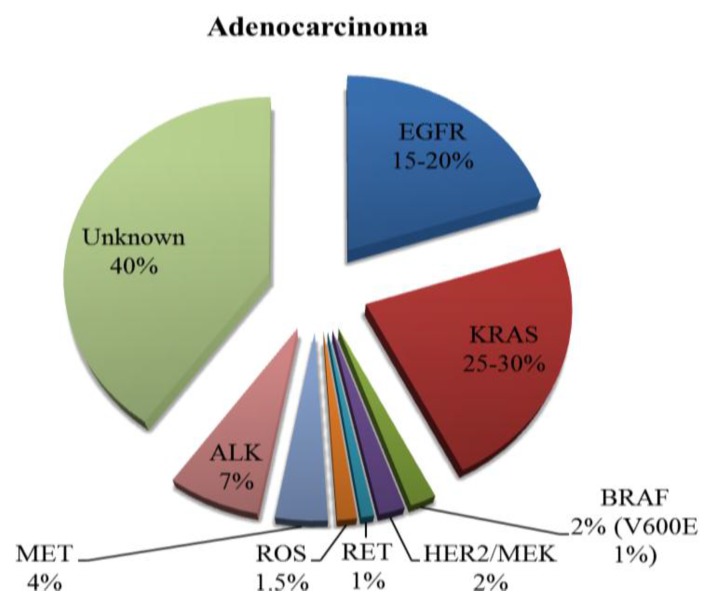
Incidence of known mutations in adenocarcinomas of the lung.

**Figure 3:**
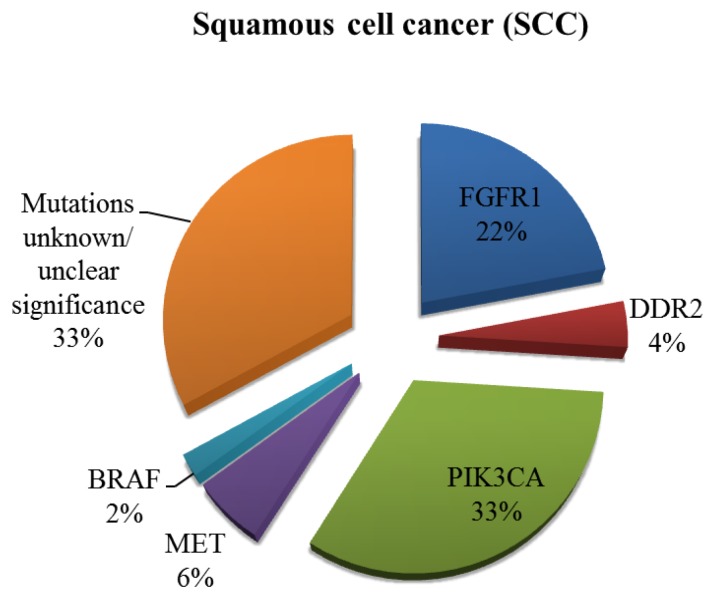
Incidence of potentially targetable driver mutations in SCC. Other mutations have been identified but their clinical significance remains questionable at present.

In recent years, there has been a rapid expansion of our knowledge regarding the molecular biology of NSCLC, which has led to the discovery of driver mutations in a significant proportion on NSCLCs. Some of these driver mutations have been identified and successfully managed with targeted therapies and others are still in development (Figure 1) [6]. The discovery of these mutations has led to a novel way of classification of NSCLC into their molecular subtypes (summarised in Figure 2 and Figure 3) [7,8].

**Figure 4:**
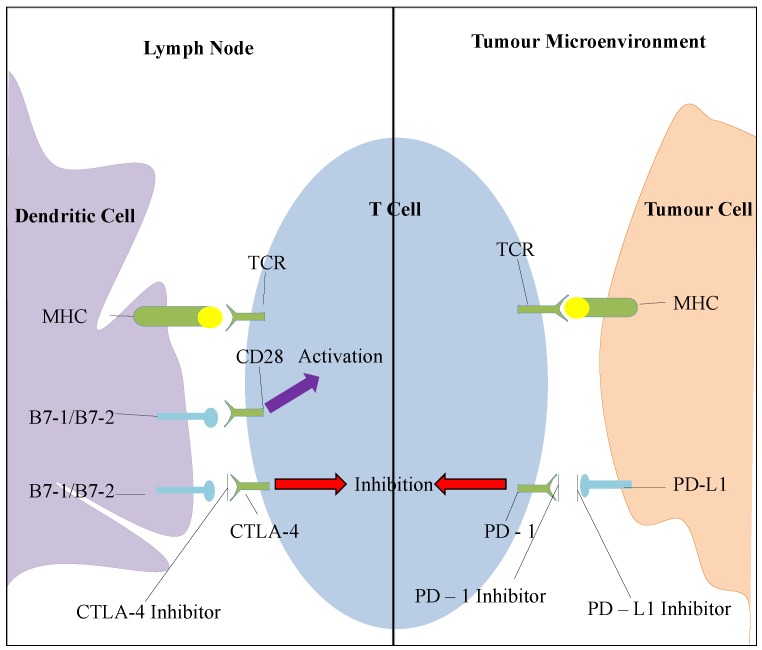
Overview of the CTLA-4 and PD-1 pathways. T cells recognize the antigens presented by the major histocompatibility complex (MHC) via their T cell receptor (TCR). This is not enough to turn on a T cell response and requires a second signal by the B7-1 (CD80) and B7-2 (CD86) costimulatory molecules. In the lymph node, where most of this interaction occurs, the molecules bind either to CD 28 and provide activation signals or to CTLA-4 and provide inhibitory signals. The blockade of CTLA-4 can therefore promote T cell activation and response. In the tumour microenvironment, long term antigen exposure leads to the expression of PD-1 receptor which inhibits T cell response when it binds to PD-L1 or PD-L2. The blockade of this inhibitory pathway results in the activation of T cells with more specificity to cancer cells.

Even more recently, the discovery of cytotoxic T lymphocyte antigen-4 (CTLA-4) and programmed death-1 receptor (PD-1) as potential targets has opened the door on immuno-oncological therapies for cancer management. CTLA-4 signalling serves to limit the initiation of T cell response in the lymph nodes and PD-1 expression is associated with limiting T cell activity in the tumour microenvironment. PD-1 interacts with the PD-L1 and PD-L2 ligands, which are expressed in tumour cells, and infiltrating immune cells thereby providing a mechanism of escape from recognition by the immune system (summarized in Figure 4) [9,10,11,12].

This review aims to summarise the current and evolving therapeutic strategies in the management of advanced NSCLC, with a focus on novel therapeutic targets, driven by particular molecular alterations.

## 2. Current Therapies Available

### 2.1. Chemotherapy

In most patients, a driver mutation cannot be identified and for those patients, chemotherapy remains the standard of care. Platinum based doublet chemotherapy has used in the 1st line setting in patients with a good performance status (ECOG 0-1). This has been established across multiple trials and meta-analyses and is the recommendation of both the American Society of Clinical Oncology (ASCO) and the European Society for Medical Oncology (ESMO). Cisplatin and carboplatin are the two platinum based compounds that have been used in combination across different studies with gemcitabine, vinorelbine, taxanes (docetaxel and paclitaxel), pemetrexed. Platinum based doublet chemotherapy combinations have shown overall response rates of 25%–35%, median progression free survival (PFS) of 4–6 months and overall survival (O.S) of 8–10 months [13,14].

Maintenance chemotherapy with pemetrexed is also recommended in non-squamous NSCLC following completion of 4–6 cycles of platinum based doublet chemotherapy. Pemetrexed is a folate antimetabolite, which inhibits enzymes used in purine and pyrimidine synthesis (thymidylate synthase, dihydrofolate reductase, glycinamide ribonucleotide formyltransferase). It has shown activity in non-squamous NSCLC across large phase III studies. It does not seem to show significant activity in SCC, possibly due to the high expression of thymidylate synthase in this histological subtype [15,16,17].

Maintenance chemotherapy in squamous NSCLC has been studied as part of trials looking at maintenance chemotherapy in all NSCLC. Single agent gemcitabine was trialed post 4 cycles of cisplatin/gemcitabine and failed to show a significant OS benefit [18]. Docetaxel was also tested as maintenance chemotherapy *vs.* use in the second line after first-line treatment. The OS in this trial was 12.3 months *vs.* 9.7 months but this was again not significant (due to the small number of patients included in the analysis) [19]. This can be a suitable approach for some patients but given the increased adverse events associated with chemotherapy, a treatment break after first-line treatment is also acceptable.

With regards to second-line chemotherapy, docetaxel was shown to improve survival *vs.* supportive care (median survival 7.0 months *vs.* 4.6 months) leading to the approval of docetaxel in this setting [20].

More recently, the compound Nab-paclitaxel (albumin-bound paclitaxel) was also shown to be effective in the first-line setting showing similar outcomes to standard paclitaxel with a better toxicity profile (PFS 6.3 *vs.* 5.8 months) [21].

### 2.2. Vascular Endothelial Growth Factor (VEGF) Inhibition

VEGF is an endothelial-cell-specific mitogen, which is a major regulator of angiogenesis in normal and malignant tissue [22]. The development of anti-VEGF therapies has also been trialled in NSCLC with some success.

Bevacizumab, a humanised monoclonal antibody to VEGF-A, was tested in combination with chemotherapy in the first-line setting. In an earlier phase study, all patients with NSCLC were included but there were significant issues with life threatening haemoptysis in a few patients (mainly those with SCC) and this led to the larger studies including non-squamous NSCLC only [23]. In a phase III study, bevacizumab was added to standard chemotherapy (carboplatin with paclitaxel) and showed a PFS and OS improvement of 6.2 months *vs.* 4.5 months and 12.3 months *vs.* 10.3 months respectively. There were some significant adverse events with bevacizumab noted in this trial however such as pulmonary haemorrhage, severe haemoptysis, pulmonary embolism [24]. Other phase III studies were conducted with bevacizumab with different outcomes. In the AVAiL study, bevacizumab was added to cisplatin and gemcitabine at 2 dose levels (7.5 or 15 mg/kg). The findings showed a small improvement in PFS (6.7 months in low dose group *vs.* 6.5 months in the high dose group *vs.* 6.1 months in the placebo group). This was statistically significant but not clinically significant given the risk of added toxicities [25]. In another study, AVAPERL, bevacizumab was added to cisplatin/pemetrexed in non-squamous NSCLC. Non-progressing patients were then randomised to maintenance bevazcizumab alone or bevacizumab plus pemetrexed. In this study, the PFS was significant (7.4 months *vs.* 3.7 months, HR 0.57) but the OS was not statistically significant despite a numerical difference (17.1 months *vs.* 13.2 months, HR 0.87) [26]. In the PointBreak study, bevacizumab was added to carboplatin/pemetrexed or carboplatin/paclitaxel and continued in combination with pemetrexed in the first group or alone in the second group. Once again, the combination of bevacizumab with pemetrexed showed no significant survival benefit (12.6 months *vs.* 13.4 months, HR 1.00) [27].

This has led to the use of bevacizumab with chemotherapy in non-squamous NSCLC in some countries although the side effect profile of the combination needs to be taken into account.

Aflibercept, a recombinant human fusion protein designed to block VEGF A and VEGF B isoforms, has also been tried in NSCLC. It was tried in the second line setting after progression post a platinum doublet chemotherapy in combination with docetaxel *vs.* docetaxel only. It failed to show any improved survival (OS 10.1 months *vs.* 10.4 months, HR 1.01) and was again associated with significant thromboembolic events [28].

Nintedanib, a tyrosine kinase inhibitor (TKI) inhibiting VEGFR, was trialled in NSCLC and showed some benefit in the second-line setting in combination with docetaxel for adenocarcinomas (OS 12.6 months *vs.* 10.3 months with docetaxel alone) although there was no OS benefit for all NSCLC (10.1 months *vs*. 9.1 months) [29]. It also did not show the same haemorrhagic side effects noted with becavizumab in the trial. It has since been approved for adenocarcinomas in the second-line setting.

More recently, the drug ramucirumab, a fully human monoclonal antibody blocking VEGFR-2, has shown some survival benefit in NSCLC. It was tested in the second-line setting in combination with docetaxel and showed a PFS and OS improvement of 4.5 months *vs.* 3.0 months and 10.5 months *vs.* 9.1 months when compared to docetaxel alone [30]. It was not associated with worse haematological adverse events and was also the first phase III trial to include SCC who made up about 25% of trial patients. This makes it the first anti-angiogenic therapy to show a benefit in SCC and will likely become the second-line standard of care in this tumour type.

### 2.3. Proven Targeted Therapies

#### 2.3.1. Epidermal Growth Factor Receptor (EGFR)

EGFR is a tyrosine kinase, which is part of the ErbB family of proteins (other known kinases in humans are Her2, Her3 and Her4). Activating mutations in the EGFR gene accounts for up to 20% of adenocarcinomas. The most common mutations identified are a deletion at exon 19 and a point mutation in exon 21 (L858R) [31,32]. These 2 mutations account for over 85% of all EGFR mutations and are more commonly associated with a specific clinical profile, which includes adenocarcinoma subtype, Asian ethnicity, female and never smoker [32,33]. These activating mutations predict response to 1st generation EGFR inhibitors whereas less common mutations such as the exon 20 insertion and the T790M mutation confer resistance to the same therapies [34,35]. EGFR mutant NSCLC is now classified as an independent subtype with its own therapies and as such all adenocarcinomas should be tested routinely to screen for mutations. SCC have very low rates of true EGFR mutations and EGFR inhibitors have not been shown to be effective in SCC overall, therefore testing for mutations is not recommended [36,37,38,39].

##### First-Generation EGFR Tyrosine Kinase Inhibitors (TKIs)

Gefitinib and erlotinib are the two approved drugs for the treatment of advanced NSCLC with EGFR mutations. Both drugs are TKIs that target the intracellular domain of EGFR through competitive reversible binding, subsequently blocking the downstream signal transduction pathways that regulate cell proliferation and apoptosis [40,41,42,43,44,45,46,47].

Both drugs have been studied in a multitude of large phase III clinical trials and shown to be superior to chemotherapy in patients with advanced NSCLC harbouring EGFR mutations [40,41,42,43,44,45]. EGFR TKIs have not been shown to be of great benefit in unselected NSCLC [46,47]. The clinical efficacy of EGFR TKIs when compared to chemotherapy is very significant and this combined with less toxicity when compared to chemotherapy has led to them being used first line for the EGFR mutant population. The main adverse events noted with EGFR TKIs are diarrhoea, nausea/vomiting and acneiform rash. The survival benefits noted across major trials is summarised in Table 1.

Invariably however, the patients develop an acquired resistance to the TKIs leading to disease progression. The most common mechanism for this is another missense mutation, which occurs in about 60% of cases. The most common of these is the T790M mutation [48].

##### Second-Generation EGFR TKIs

The emergence of resistance through mutations led to the development of second-generation TKIs. These TKIs are irreversible pan-ErbB inhibitors and have demonstrated activity in the T790M mutations in animal models [49,50]. The three main second-generation TKIs with clinical data are afatinib, dacomitinib and neratinib (results summarised Table 1).

Afatinib binds irreversibly to EGFR, HER2, HER4 and also to EGFR receptors carrying the T790M mutation, which could provide an avenue to overcome resistance [51]. In a phase III study in the first line setting, afatinib was shown to be superior to chemotherapy in EGFR mutant NSCLC with a progression free survival (PFS) of 11.1 months *vs.* 6.9 months in the chemotherapy group (HR 0.58) [52]. In a single arm phase II study in second line therapy in EGFR TKI naïve patients with EGFR mutant NSCLC, the PFS was shown to be about 14 months [53]. In the second line setting post first-generation TKI, it has been tested in a phase II and a phase III study and shown a small benefit with PFS of 4.4 months and 3.3 months respectively although this did not translate to overall survival benefit in the phase III study [54,55]. Of note, across all trials using afatinib, 12% of patients harbored an uncommon mutation in EGFR and most of those responded to therapy [56]. It is reasonably well tolerated with diarrhoea and rash as the main significant adverse events.

Dacomitinib binds irreversibly to EGFR, Her2 and Her4. It was trialled in the phase III setting in the second/third line post chemotherapy against erlotinib and failed to show a survival benefit at the cost of greater toxicities [57]. In another phase III study, it was trialled against placebo in the third line setting post EGFR TKI and again failed to show a survival benefit [58]. It is important to note that both trials included non-mutants as well as mutant EGFR cancers. A phase III study ARCHER 1050 is currently running and will compare dacomitinib to gefitinib in the first line setting in EGFR mutant patients.

Neratinib, another irreversible inhibitor of EGFR, Her2 and Her4 was trialled in the phase II setting but failed to show any survival benefit mainly due to its significant toxicity profile (mainly diarrhoea), which required multiple dose adjustments [59].

The role of second-generation EGFR TKIs at present seems limited in the acquired resistance setting but may offer better options in first line therapy although this needs to be balanced with the toxicities of the drugs, which have been more prominent than the first-generation TKIs [57,59].

**Table 1:** Trials showing the efficacy of EGFR TKI in EGFR mutant advanced NSCLC.

First Generation EGFR TKI (Erlotinib, Gefitinib)-First-Line Studies						
Trial	Population	Number	Agent (A)	Comparator (C)	Median PFS A *vs.* C (Months)	HR
**Mok *et al.* (2009) [40] Phase III**	Adenocarcinoma, Asian, never or light smokers	1217	Gefitinib	Carboplatin, pacltaxel	9.8 *vs.* 6.4	0.48
**Maemondo *et al.* (2010) [41] Phase III**	EGFR mutant	230	Gefitinib	Carboplatin, paclitaxel	10.8 *vs.* 5.4	0.30
**Mitsudomi *et al.* (2010) [42] Phase III**	EGFR mutant	172	Gefitinib	Cisplatin, docetaxel	9.2 *vs.* 6.3	0.489
**Han *et al.* (2012) [43] Phase III**	Adenocarcinoma, Asian, never or light smokers	309	Gefitinib	Cisplatin, gemcitabine	5.8 *vs.* 6.4	1.198
		42 EGFR +ve			8.0 *vs.* 6.3	0.544
**Zhou *et al.* (2011) [44] Phase III**	EGFR mutant	154	Erlotinib	Carboplatin, gemcitabine	13.1 *vs.* 4.6	0.16
**Rosell *et al.* (2012) [45] Phase III**	EGFR mutant	173	Erlotinib	Platinum doublet	9.7 *vs.* 5.2	0.37
**Second Generation EGFR TKI (Afatinib, Dacomitinib, Neratinib)**						
**Sequist *et al.* (2013) [52] Phase III**	EGFR mutant- 1st line setting	345	Afatinib	Cisplatin, pemetrexed	11.1 *vs.* 6.9	0.58
**Wu *et al.* (2014) [60] Phase III**	EGFR mutant- 1st line setting	364	Afatinib	Cisplatin, gemcitabine	11.0 *vs.* 5.6	0.28
**Yang *et al.* (2012) [53] Phase II**	EGFR mutant- 2nd line with no prior EGFR TKI	129	Afatinib	Single arm	14.0	NA
**Miller *et al.* (2012) [54] Phase IIb/III**	EGFR mutant- after treatment with EGFR TKI	585	Afatinib	Placebo	3.3 *vs.* 1.1	0.38
**Katakami *et al.* (2013) [55] Phase II**	Clinical post 1st line EGFR TKI, 72.6% mutant	62	Afatinib	Single arm	4.4	NA
**Ramalingam *et al.* (2014) [57] Phase III**	All NSCLC post progression with chemotherapy	878	Dacomitinib	Erlotinib	2.6 *vs.* 2.6	1.022
**Ellist *et al.* (2014) [58] Phase III**	All NSCLC post progression with chemotherapy and EGFR TKI	720	Dacomitinib	Placebo	2.7 *vs.* 1.4	0.66
**Sequist *et al.* (2010) [59] Phase II**	All NSCLC	167	Neratinib	Single arm	3.7 (Severe diarrhoea)	NA
**Third Generation EGFR TKI (Rociletinib, AZD9291)**						
**Sequist *et al.* (2015) [61] Phase I/II**	EGFR mutant post progression on EGFR TKI	130	Rociletinib	Single arm	13.1	NA
**Janne *et al.* (2015) [62] Phase I/II**	EGFR mutant post progression on EGFR TKI	253	AZD9291	Single arm	8.2	NA

##### Third-Generation EGFR TKIs

This class of TKIs irreversibly inhibits mutant EGFR, in particular T790M with minimal activity on the other members of the ErbB family. One of these agents, rociletinib (CO-1686) showed objective response rates of 59% in patients with T790M mutation positive EGFR mutant NSCLC who had progressed on an EGFR inhibitor in a phase I/II trial. It also showed a response rate of 29% in T790M negative patients [61]. In a similar study, the drug AZD9291 showed similar response rates with 61% objective response in T790M positive patients and 21% in T790M negative patients with a PFS of 9.8 months in the T790M positive group and 2.8 months in the T790M negative group [62]. Larger trials are now underway but these agents could be the potential second line TKI after resistance to 1st line EGFR inhibition. It also means that patients with an EGFR mutation should be re-biopsied on progression to determine the best next line of therapy.

##### Dual Inhibition

A recent phase Ib study combining afatinib with cetuximab (an anti-EGFR monoclonal antibody) was conducted based on pre-clinical data [63]. It was trialled in patients who had progressed on a first line EGFR TKI with EGFR mutant NSCLC. It showed a response rate of 29%, which was similar in T790M positive and negative cohorts and showed a PFS of 4.7 months. However there were significant side effects in 99% of patients and grade 3 toxicities in 44%, which would limit the use of such a combination [64].

##### Treatment Paradigm Following First-Line EGFR TKI

The approach following progression on a first-line EGFR TKI has been rapidly evolving together with the novel drug therapies and advances in the understanding of EGFR mutated lung cancers. Initially, once a patient progressed on an EGFR TKI, the only option was to change to platinum based doublet chemotherapy. However, when ceasing the EGFR TKI upon progression, a proportion of patients would have a rapid disease flare within a few days [65]. This has led to different trials aiming to maintain disease control and prevent these flares.

In the ASPIRATION study, erlotinib was continued beyond progression on imaging as per RECIST. In the patients who continued beyond progression (at investigator’s discretion), there was a difference in PFS of 3.7 months [66]. The survival data from this study is not yet available but it demonstrates that in some patients, there is a potential benefit in continuing treatment despite radiological progression which could be important when planning for further therapies or clinical trials in order to minimize the risk of disease flares.

The combining of chemotherapy to an EGFR TKI following progression after first-line EGFR inhibition was also tested in a trial setting (IMPRESS study). Patients who had progressed on first-line gefitinib were randomised to continuing gefitinib with platinum doublet chemotherapy or having chemotherapy alone. This was a negative study with the PFS identical for both groups (5.4 months) and although the OS data is not yet available, this has shown that there would be no benefit to persisting with the EGFR inhibitor in combination with chemotherapy [67].

These studies help determine the best course for EGFR mutation positive patients following progression on first-line EGFR TKIs. The TKI could be continued beyond progression whilst a clinical benefit is being derived but should be ceased prior to switching to chemotherapy. This can be important given the emerging role for biopsy on progression, as patients could remain on their first-line EGFR TKI whilst waiting for biopsy and determining eligibility for second-line therapies or clinical trials. With the development of third-generation EGFR TKIs however, they will likely become the accepted second-line therapy.

##### EGFR Inhibition in SCC

The original discovery of EGFR as possible biomarker led to the FLEX study which looked at the addition of cetuximab, a monoclonal antibody to EGFR, to 1st line chemotherapy [68]. This study showed a modest survival benefit in patients with high EGFR expression and as this was shown in about 7% of SCCs, this was further studied in another trial involving another agent blocking the EGFR pathway. This agent, necitumumab, is a humanised monoclonal antibody that is designed to block the ligand binding site of the human epidermal growth factor receptor 1 (EGFR). In a phase III study (SQUIRE study) it was compared to placebo when added to first-line chemotherapy in patients with advanced SCC of the lung. It did show a modest survival benefit (median OS 11.9 months *vs.* 9.9 months, HR 0.84) but this came at a cost of a significant increase in serious adverse events [69]. Another phase 3 study also compared the use of afatinib *vs.* erlotinib in the second-line setting (LUX-Lung 8 study). This study was also positive with a modest PFS of 2.4 months *vs.* 1.9 months (HR 0.82) and a median OS of 7.9 months *vs.* 6.8 months (HR 0.81) [70]. Once again there were significant adverse events such as diarrhea, rash and stomatitis. Whilst it is encouraging that there was a statistical improvement in both trials, the clinical relevance of these studies remains to be seen given the toxicities involved for a small survival improvement.

#### 2.3.2. Anaplastic Lymphoma Kinase (ALK)

Rearrangements in the ALK gene are found in 5%–7% of lung adenocarcinomas. A fusion gene involving ALK and the Echinoderm Microtubule Associated Protein-Like 4(EML4) gene results in the constitutive activity of the ALK pathway [71]. ALK positive (defined as a rearrangement in the ALK gene) lung cancer patients are usually younger, non-smokers and adenocarcinoma histology [72].

Crizotinib is a small molecule ALK TKI that leads to arrest of the cell in the G1-S phase and induction of apoptosis [73]. In the first phase I study, it demonstrated an objective response rate of 60.8% and a median PFS of 8.1 months [74,75,76]. Common toxicities reported with crizotinib include visual disturbances, nausea, vomiting, diarrhoea, oedema and development of benign renal cysts. More serious side effects like fatal hepatotoxicity and pneumonitis are uncommon. A phase II study demonstrated objective response rates of 59.8% and a median PFS of 8.1 months [77]. This led to two phase III studies. In the first, crizotinib was compared to single agent pemetrexed or docetaxel in the second line setting after platinum based chemotherapy. Crizotinib showed a higher response rate (65% *vs.* 20%) and a superior PFS (7.7 months *vs.* 3.0 months, HR 0.49) [78]. In the second study, crizotinib was compared to platinum based doublet chemotherapy in the first line setting. In this study, crizotinib was once again superior with the objective response rate being 74% *vs.* 45% with chemotherapy; the PFS was in favour of crizotinib (10.9 months *vs.* 7 months, HR 0.45) [79]. These have led to the approval of the use of crizotinib in first line ALK-positive advanced NSCLC.

Additionally, crizotinib is an inhibitor of MET [80] and ROS1 [81] tyrosine kinases and has therapeutic implications in NSCLC exhibiting these mutations. Unfortunately, it appears crizotinib has a poor blood-brain barrier penetration leading to a high rate of relapse in the central nervous system (CNS); in the early phase studies of crizotinib, CNS recurrence was the most common cause of progressive disease [74,75,76,77]. There has also been the emergence of resistance to crizotinib therapy through various mechanisms. The most common are secondary mutations in the ALK kinase domain (L1196M and G1269A are the most common of those), which have been shown in 29% of cases. Other causes included amplification of the rearranged ALK locus, activation of alternative tyrosine kinases but in most cases the cause is unknown at present [82].

##### Second-Generation ALK Inhibitors

Resistance to crizotinib has led to the development of novel ALK inhibitors and there are currently multiple agents being trialed in the phase I setting, with ceritinib and alectinib having the most clinical data at this point in time.

Ceritinib, a novel ALK TKI, was tested in a phase I study in patients with advanced ALK-positive NSCLC (mixed group of patients who had progressed post crizotinib and ALK TKI naïve) and showed a response rate of 58% (similar across both groups) and a median PFS of 7.0 months. Interestingly it showed activity in ALK kinase mutations, ALK amplifications as well as other causes of resistance [83]. In a retrospective analysis, sequential treatment with ceritinib immediately following progression on crizotinib showed a combined median PFS of 17.4 months and median overall survival (OS) of 49.4 months. Again the cause of resistance to crizotinib did not affect treatment response to ceritinib [84].

Alectinib, a more potent ALK TKI, was trialed in a phase I/II study in ALK-positive advanced NSCLC. It showed response rates of 93.5% and median PFS has not yet been reached. At the latest update, patients had been followed up beyond 10.3 months indicating better and more durable responses than crizotinib although the data has not matured yet [85,86]. Furthermore, it appears alectinib has greater CNS penetration having shown responses in the CNS even after crizotinib and ceritinib in a few cases [87]. Table 2 summarises the outcomes from major trials in ALK-positive NSCLC to date.

Multiple larger studies are underway to confirm these benefits as well as earlier phase studies in newer ALK TKI compounds.

##### Other Targets

Heat Shock Protein 90 (Hsp 90) is a protein involved in regulating the correct folding, stability and function of numerous proteins involved in normal cellular function as well as tumourigenesis [88]. In a small number of patients, ganetespib, a Hsp 90 inhibitor, showed activity in ALK-positive advanced NSCLC in ALK TKI naïve patients as well as in a patient following treatment with crizotinib [89]. Trials are underway to combine these inhibitors with ALK inhibitors.

**Table 2:** Trials showing efficacy of ALK TKI in ALK positive advanced NSCLC.

First Generation ALK TKI-Crizotinib						
Trial	Population	Number	Agent (A)	Comparator (C)	Median PFS A *vs.* C (Months)	HR
**Kim *et al.* (2012) [77] Phase II**	ALK positive post 1st line chemotherapy	439	Crizotinib	Single arm	8.5	NA
**Shaw *et al.* (2013) [78] Phase III**	ALK positive post 1st line chemotherapy	347	Crizotinib	Pemetrexed or docetaxel	7.7 *vs.* 3.0	0.49
**Solomon *et al.* (2014) [79] Phase III**	ALK positive- 1st line	343	Crizotinib	Platinum, pemetrexed	10.9 *vs.* 7.0	0.45
**Second Generation ALK TKI (Ceritinib, Alectinib)**						
**Shaw *et al.* (2014) [83] Phase I**	ALK positive (68% progressed on Crizotinib)	130	Ceritinib	Single arm	7.0 overall, 10.4 for ALK inhibitor naïve, 6.9 in prev. treated	NA
**Seto *et al.* (2013) [85,86] Phase I/II**	ALK positive- 1st line setting	58	Alectinib	Single arm	Not yet reached >10.3	NA

### 2.4. Potential Targets under Study

#### 2.4.1. ROS1

Aberrant fusions involving the ROS1 gene occur in about 1.5% of lung adenocarcinomas. The clinical presentations are very similar to ALK positive lung cancers as ROS1 positive lung cancers tend to affect younger patients, never smokers and adenocarcinoma differentiation [90]. Crizotinib, an ALK inhibitor has demonstrated activity in this subset in a phase I study involving 50 patients [91]. In this trial, the response rate was 72% and median PFS was 19.2 months, which is very impressive for a NSCLC study. It will be interesting to see if newer, more potent ALK inhibitors with ROS1 activity can provide an ever better response in this subset of NSCLC.

#### 2.4.2. Mesenchymal-Epithelial Transition (MET)

Amplification of the c-MET gene is present in about 4% of lung adenocarcinomas and is usually associated with a poorer prognosis [92]. Crizotinib as well as cabozantinib, a TKI which inhibits c-MET, VEGFR2, have shown some early activity in lung adenocarcinomas with MET mutations [93]. Of note, there is currently no evidence of MET inhibition in SCC which carries a 6% mutation rate.

#### 2.4.3. Rearranged during Transfection (RET)

RET is a fusion gene involving kinesin family member 5B (KIF5B) as a fusion partner, present in up to 1.7% of patients with lung adenocarcinomas [94]. An early phase study is showing activity with cabozantinib, which can also block RET [95] as well as ROS-1. Further studies with this drug are now underway.

#### 2.4.4. BRAF Mutation

BRAF is a proto-oncogene, which can be mutated in a wide range of cancers although it is more common in melanoma. Of the known mutations, the most common and targetable mutation is the V600E variant [96]. It occurs in about 2% of lung adenocarcinomas and 2% of SCC, with the V600E mutation pattern noted in about 1% of lung adenocarcinomas. A phase II study is currently ongoing with the combination of the BRAF and MEK inhibitors dabrafenib and trametinib which has shown some activity so far [97].

### 2.5. Targets with No Currently Effective Therapy

#### 2.5.1. Kirsten Rat Sarcoma 2 Viral Oncogene Homolog (K-RAS) Mutation

K-RAS is part of a family of GTPase molecules responsible for the transducing of growth signals from a wide range of tyrosine kinase receptors including EGFR. Activating mutations in this gene accounts for up to 30% of all mutations in adenocarcinomas of the lung [98]. The presence of K-RAS mutation is usually mutually exclusive with EGFR or ALK mutations and would therefore predict resistance with the therapies targeting these [99].

Selumetinib, an inhibitor of MEK1/MEK2, has been studied in combination with docetaxel in second-line in K-RAS mutant NSCLC in a phase II study *vs.* docetaxel alone. It did manage to show an improvement in median PFS (5.3 months *vs.* 2.1 months, HR 0.58). However, this came at a cost of significant adverse events with 82% of patients in the selumetinib arm having grade 3 or higher adverse events [100]. The role of K-RAS blockade is still being investigated at present.

#### 2.5.2. Insulin-Like Growth Factor 1 Receptor (IGFR1)

IGFR1 is a mediator of cellular proliferation frequently overexpressed in NSCLC. Figitumumab, a fully human anti-IGF-1R monoclonal antibody, was trialled in a phase II setting in combination with a standard chemotherapy doublet and early results suggested an improvement in SCC lung cancer. This led to a phase III trial in advanced lung SCC that was stopped at the first interim analysis due to futility. A reassessment of the phase II data showed no improvement in any subtype and this has led to a retraction of the study [101,102]. There is no current ongoing development of IGFR1 inhibitors at present.

#### 2.5.3. Fibroblast Growth Factor Receptor 1 (FGFR1)

FGFR1 amplification is common in lung SCC occurring in up to 22% of cases [8]. It is also associated with poor survival and extensive smoking history [103]. AZD4547, a potent and selective FGFR 1–3 inhibitor, was tested in advanced lung SCC in the phase I setting but failed to demonstrate any response and was closed early [104].

#### 2.5.4. Discoidin Domain Receptors (DDR)

DDRs are receptor tyrosine kinases that belong to the EGFR family. Mutations are found in about 4% of lung SCC. Dasatinib, an oral Bcr-Abl tyrosine kinase inhibitor used in chronic myelogenous leukaemia (CML), was thought to be a potential inhibitor of DDR but trial with this drug was abandoned due to the difficulty in recruiting patients. No other drugs are currently being trialled.

### 2.6. Immunotherapy in NSCLC

#### 2.6.1. CTLA-4 Inhibition

Ipilimumab, a humanised monoclonal antibody to CTLA-4, facilitates stimulation of T-cell activity and potentially enhance anti-tumour responses. This has been demonstrated in melanoma in clinical practice [105]. This positive study led to the trial of ipilimumab in NSCLC. A phase II study in the first-line setting compared chemotherapy (carboplatin, paclitaxel) to chemotherapy with 4 concurrent cycles of ipilimumab to 2 cycles of chemotherapy followed by 4 cycles of ipilimumab with chemotherapy (phased). The median immune related PFS was 5.7 months for the phased group (HR 0.72), 5.5 months for the concurrent group (HR 0.81) and 4.6 months for the chemotherapy alone group [106]. The median OS for the phased group was 12.2 months which is similar to the bevacizumab data. Based on this, a phase III study is currently recruiting. Ipilimumab is also associated with significant adverse events due to T-cell activation and proliferation, therefore any benefit needs to be assessed in function of the risk of harm involved.

#### 2.6.2. PD-1 Pathway Inhibition

A novel immune checkpoint pathway of relevance in NSCLC is that between the PD-1 receptor (expressed on activated T-Cells) and its ligands PD-L1 (programmed death 1 ligand) and PD-L2 (programmed death 2 ligand) which are produced by stromal and tumour cells [107]. Its role is to protect tissues by shielding them from T-cell activity and in tumour cells expressing PD-1, help tumour cells escape the immune system. Inhibition of the PD-1 pathway has been studied with regards to melanoma where it has shown a survival advantage [108]. The expression of PD-L1 has been shown in multiple different cancer types including NSCLC although it is unclear if it can be used as a biomarker currently.

Nivolumab, a human monoclonal antibody to PD-1, prevents the activation of the PD-1 pathway. In early trials, it showed efficacy in previously treated NSCLC [109], which led a phase III study in second-line compared to docetaxel in advanced SCC. This landmark study, CheckMate 017, was stopped early after an independent data monitoring committee concluded that the study had met its endpoint in demonstrating a superior survival benefit for nivolumab when compared to docetaxel (median PFS 3.5 months *vs.* 2.8 months, HR 0.62). The overall survival was 9.2 months in the nivolumab group and 6.0 months in the docetaxel group (HR 0.59). Additionally, nivolumab had a better safety profile with only 7% having grade 3 or higher adverse events compared to docetaxel, which had a 55% incidence [110]. The benefit was independent of PD-L1 expression and will soon become the standard of care second-line therapy.

Another phase III study involving nivolumab, CheckMate 057, has evaluated the same study design in advanced non-squamous NSCLC. In this study, nivolumab was again shown to be superior to docetaxel with median OS being 12.2 months *vs.* 9.4 months (HR 0.73). Median duration of response was 17.2 months in the nivolumab group *vs.* 5.6 months in the docetaxel group. Interestingly, PD-L1 expression in this study was predictive of better response to nivolumab although overall the patients in the study group did better than those in the docetaxel arm. Nivolumab once again had fewer serious adverse events than docetaxel [111]. Until more information is available about biomarkers, these findings will hopefully lead to the use of nivolumab in the second-line setting for all NSCLC.

Pembrolizumab, another PD-1 blocking humanised monoclonal antibody, has also shown activity in the phase I setting (median duration of response 12.5 months, median OS 12.0 months) [112].

Further studies of PD-1 inhibitors are ongoing as well as PD-L1 inhibitors (such as atezolizumab) and combination of CTLA-4 and PD-1 inhibitors.

Another positive aspect of checkpoint inhibitors is that of durable responses in patients who responded to the therapies. Although this happens in only around 25% of the patients, the prolonged disease control is still very exciting. However, more work is required to identify biomarkers, which could identify patients who would most likely benefit from the treatment. As the data from the ongoing studies mature, we will obtain more information about which cancers would benefit the most from these therapies and also how durable responses impact on overall survival.

### 2.7. Stem Cell Inhibitors

There is accumulating evidence supporting the cancer stem cell hypothesis. This stipulates that a subset of cancer cells retain the property to self-renew and give rise to differentiated progeny. These cells, called cancer stem cells (CSCs) drive tumour growth and metastasis and are more likely to be resistant to traditional anti-cancer therapies such as chemotherapy or radiation [113]. A pathway that appears to be essential for CSCs is the Notch pathway, which is made of 4 Notch receptors (1–4) and 5 ligands, Jagged (1,2) and Delta-Like Ligand (DLL 1,3 and 4). Demcizumab is a humanised monoclonal antibody that blocks DLL4. In a phase Ib study, demcizumab showed some activity in combination with carboplatin and pemetrexed in non-squamous NSCLC in the first line setting [114]. This has led to a phase II study, which is currently recruiting.

### 2.8. Other Potential Biomarker Based Clinical Trials

Other biomarkers have also been devised to assist in predicting response to the available therapies and help in improving patient selection as well as directing further lines of therapy.

One such biomarker is VeriStrat. This is a proteomic serum test which classifies patients in a good or poor group and predicts response to EGFR TKIs [115]. In a phase III trial in the second line setting, patients were randomized according to the VeriStrat status and then randomized to either erlotinib or chemotherapy (pemetrexed or docetaxel). Overall survival according to the treatment groups was not significantly different (7.7 months in erlotinib group *vs.* 9.0 months in chemotherapy group, HR1.22). However, the patients in the good classification group had a median OS of 11.0 months with erlotinib and 10.9 months with chemotherapy (HR1.06) compared with those in the poor classification group (3.0 months with erlotinib *vs.* 6.4 months with chemotherapy, HR 1.72) [116]. The use of this proteomic test could certainly be helpful as a prognostic tool for patients given the survival difference between the two groups as well as help determine the best next line of therapy. The predictive value of the test for response to EGFR TKIs is interesting but probably not as important a marker in this setting as a known EGFR activating mutation. Nevertheless, it could be helpful in situations where there is insufficient tissue as a method to determine if a patient would benefit from the EGFR TKI. Further studies to evaluate the practical use of this test are required.

With regards to helping with predicting response to chemotherapy, other biomarkers have been previously identified. These include excision repair cross-complementing 1 (ERCC1) gene [117], ribonucleotide reductase subunit M1 (RRM1) [118] and class III beta tubulin (β-tubulin III) [119]. High levels of ERCC1, RRM1 and β-tubulin III have been associated with resistance to cisplatin, gemcitabine and taxanes respectively. The feasibility of using ERCC1 and RRM1 expression was tested in a single arm study and shown to be a feasible test to determine which chemotherapy combination to give to patients with advanced NSCLC [120]. A prospective trial comparing stratification by expression of ERCC1, RRM1 and β-tubulin III into standard chemotherapy or chemotherapy according to their biomarker expression was also performed but this failed to show any significant OS benefit for chemotherapy selection via biomarker (Median OS 13.5 months in the study group *vs.* 12.5 months in the control group) [121]. Currently there is no role for using biomarkers to select chemotherapy agents outside of a clinical trial setting.

### 2.9. Special Considerations for Elderly Patients

The median age of diagnosis of lung cancer is 71 years of age, making elderly patients a significant proportion of NSCLC sufferers [5]. Given the potential toxicities of all the therapies discussed previously, the approach when treating elderly patients needs to take them into account as they are not included in most clinical trials.

There has been evidence to support the use of platinum based doublet chemotherapy even in elderly patients. A retrospective analysis of outcomes by age of a phase III trial using platinum based doublet chemotherapy in the first line setting showed that the adverse events were similar in all age groups with the exception of younger patients having more nausea. The OS was not statistically different between patients <70 years (8.6 months) and those ≥70 years (7.9 months) [122]. Another phase III trial in patients aged 70–89 demonstrated a superiority of platinum based doublet chemotherapy over single agent chemotherapy (Median OS 10.3 months *vs.* 6.2 months, HR 0.64) although this did come with increased toxicities [123]. These studies both show that platinum based doublet chemotherapy is preferred in elderly patients; however it is important to note that these were fit elderly patients with an excellent performance status and may not be easily adaptable in the clinic setting where the patients may have a worse performance status or significant comorbidities. Single agent chemotherapy approaches have also been successfully trialed in elderly populations. Vinorelbine, gemcitabine, weekly paclitaxel and docetaxel have all shown significant improvement in quality of life and survival [124,125,126,127]. This approach may be useful in patients where platinum based doublet chemotherapy is not adequate and would be a reasonable alternative in elderly patients with comorbidities.

In patients harboring an EGFR mutation, there is significant data from multiple trials supporting the efficacy of either gefitinib or erlotinib in an elderly population [128,129,130,131]. In the majority of the patients involved in these trials, one or more dose reductions were required in order to manage toxicities such as rash, diarrhea or elevated transaminase level. Gefitinib is also often preferred to erlotinib as first line therapy in the elderly as it is thought to be less toxic than erlotinib based on a retrospective analysis [132].

There is also some evidence for the use of EGFR TKIs in the first line setting for patients with a poor performance status. In a small study, 30 patients with an ECOG of 2–4 with an EGFR mutation were treated with gefitinib with a median OS of 17.8 months [133]. This highlights the better tolerability of EGFR TKIs when compared to platinum based doublet chemotherapy outlining their use in elderly patients as well as patients with a poor performance status. Furthermore, in the TOPICAL trial, patients deemed unsuitable for chemotherapy (either due to poor performance status or multiple comorbidities) were treated with either erlotinib or placebo. This identified the presence of an early rash (within 28 days) as an adverse event to be predictive of response to erlotinib. It is important to specify that the patients were not selected for EGFR mutations on this study and the activating mutations were only detected in 7% of patients. The median overall survival was 6.2 months (HR0.76) for patients receiving erlotinib and developed an early rash, 2.9 months (HR1.30) for those receiving erlotinib without a rash and 4.1 months in the placebo group [134].

There is very little data available currently with regards to the new therapeutic options and their use in elderly patients. It is likely that they will benefit from these therapies but their use needs to be balanced with regards to their toxicities.

The importance of adequate supportive care in the management of advanced NSCLC also needs to be highlighted. In a study performed at the Massachusetts General Hospital, early referral to a specialized palliative care service was associated with greater symptom control benefit as well as a survival benefit (11.6 months *vs.* 8.9 months) [135]. This demonstrates the importance of good symptom control in patients with advanced lung cancer and can be used in combination with systemic therapies to achieve the greatest symptomatic and survival benefit.

## 3. Conclusions

The landscape of advanced NSCLC therapies is rapidly evolving. The concept of personalised medicine, where patients are selected based on their histological subtypes and molecular profiles, is increasingly being practiced. The testing for driver mutations in non-squamous NSCLC is becoming more and more essential as the therapies for mutation driven cancers are now quite different to non-mutated cancers. In most cases however, targetable driver mutations are not found and for these patients, chemotherapy remains the standard of care.

Lung adenocarcinomas have shown more driver mutations that can be successfully targeted such as EGFR, ALK, ROS1. The average survival showed with those agents offer a more positive outlook on a cancer that is still the leading cause of cancer death worldwide. Furthermore, it is becoming apparent that biopsy on progression is more and more useful to determine the best course of further therapies for patients with known driver mutations. This creates a new set of challenges however given the difficulty in obtaining new biopsies (lesions can be difficult to access) as well as the heterogeneity of lung cancers. As outlined in the two CheckMate studies discussed previously, PD-L1 expression was useful in one study but not the other [110,111]. The reasons for this could be because it’s not the correct biomarker or because the assay method is not reliable. It could also be due to differential expression of biomarkers at different sites of disease. Further research into other methods of obtaining reliable information about the change in the biology of lung cancers on progression is needed. This could include circulating tumour DNA assays from peripheral blood or functional imaging guided biopsies (e.g., PET guided biopsies).

**Figure 5:**
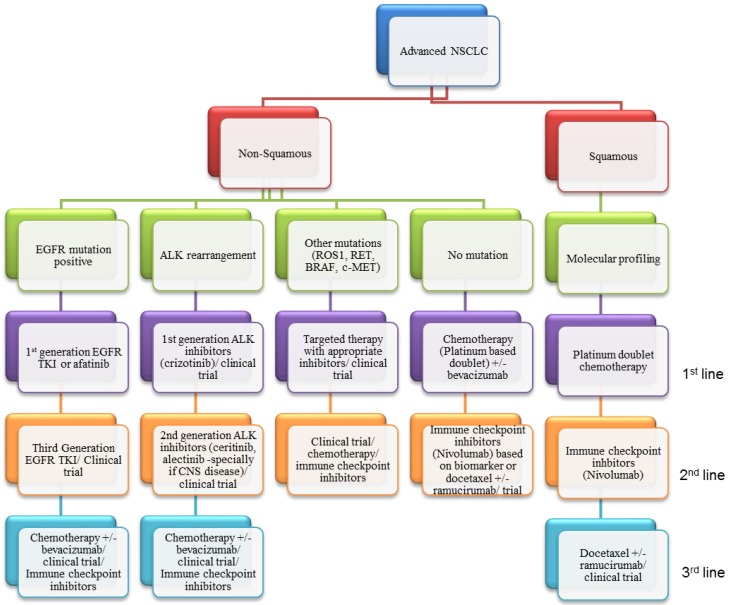
A likely algorithm for incorporating different therapies into the management of NSCLC based on current evidence. Profiling of all NSCLC including histological typing, molecular profiling and genetic analysis is of paramount importance to identify the patients who will benefit from targeted therapies. Based upon the presence of driver mutations, patients will go onto the recommended therapy for their molecular subtype. Biopsy upon progression is also likely to become increasingly common in order to identify resistance mechanisms and rationalise further lines of therapy. For patients with no actionable mutation, chemotherapy, VEGF inhibition and immunotherapy will offer the best therapeutic option. Better predictive biomarkers are required however to maximise the benefit of available therapies.

SCCs of the lung however, have not shown a great response to targeted agents until now. However, the arrival of checkpoint inhibitors has the scope to dramatically change expectations with the positive studies in SCC as well as positive findings in adenocarcinomas. It is likely that immunotherapy will become standard of care for second-line treatment although it is yet unclear how great the impact on overall survival will be. There is also still a large amount of work to be done with regards to the identification of predictive biomarkers for response and clinical outcome. Further studies are also needed in order to determine the best combination of checkpoint inhibitors and whether they could be used in combination with chemotherapy or even targeted agents. The long term responses seen in other cancers such as melanoma are indeed very exciting but time will tell if similar responses can be seen in patients with advanced NSCLC. The current classification and available therapies are summarized in Figure 5.

It is imperative that better biomarkers are developed in order to further the concept of cancer treatment personalisation as more and more active agents are being discovered and being brought into clinical practice.
